# The SystemCHANGE™ intervention improves immunosuppressive medication-taking habit: a secondary analysis of the MAGIC studies

**DOI:** 10.3389/ti.2026.16252

**Published:** 2026-09-01

**Authors:** Cynthia L. Russell Lippincott, Antoine Pironet, Yaprak Sarigol Ordin, Rebecca Bartlett Ellis, Buket Celik, Eda Ayten Kankaya, Mary Beth Stephens, Serkan Yildiz, Preethi Yerram, Wuraola Awopetu, Mark Wakefield, Elizabeth Henderson

**Affiliations:** 1 School of Nursing and Health Studies, University of Missouri-Kansas City, Kansas City, MO, United States; 2 AARDEX Group, Seraing, Belgium; 3 Dokuz Eylul University, Izmir, Türkiye; 4 Indiana University School of Nursing, Indiana University, Indianapolis, IN, United States; 5 Transplant Department, University of Missouri Health Care, Columbia, MO, United States; 6 Medicana Hospital, Istanbul, Türkiye; 7 Division of Nephrology, Department of Medicine, University of Missouri-Columbia and Harry S. Truman Hospital, Columbia, MO, United States; 8 Undergraduate Program, University of Missouri-Kansas City, Kansas City, MO, United States; 9 Division of Urology, University of Missouri Health Care, Columbia, MO, United States; 10 Undergraduate Program, Saint Louis University, St. Louis, MO, United States

**Keywords:** adults, habit strength, kidney transplantation, medication adherence, secondary analysis

## Abstract

Habit impacts healthy behaviors. The SystemCHANGE™ intervention supports associating medication taking with existing habits or creating new habits to improve medication adherence. This secondary analysis of United States and Türkiye MAGIC study data examines differences in habit between SystemCHANGE™ and attention control groups, differences in habit between countries, and habit as a relationship mediator between the SystemCHANGE™ intervention and immunosuppressive medication adherence in adult kidney transplant recipients. The Medication Event Monitoring System measured medication adherence. Objective habit index was computed during screening, intervention, and maintenance. During the SystemCHANGE™ intervention, habit strength was higher than during the screening and maintenance periods in both groups (mean difference 0.042, 95% confidence interval (CI): 0.004 to 0.080, p = 0.030). Habit index was lower for US participants compared to Turkish participants (mean difference −0.195, 95% CI: −0.300 to −0.089, p < 0.001). Habit was a mediator of the SystemCHANGE™ intervention on medication adherence (indirect effect was 0.094, 95% CI: 0.080 to 0.114, p < 0.001). The SystemCHANGE™ intervention exerted an impact on habit in two different populations which then had a positive impact on medication adherence.

## Graphical Abstract

**Figure:**
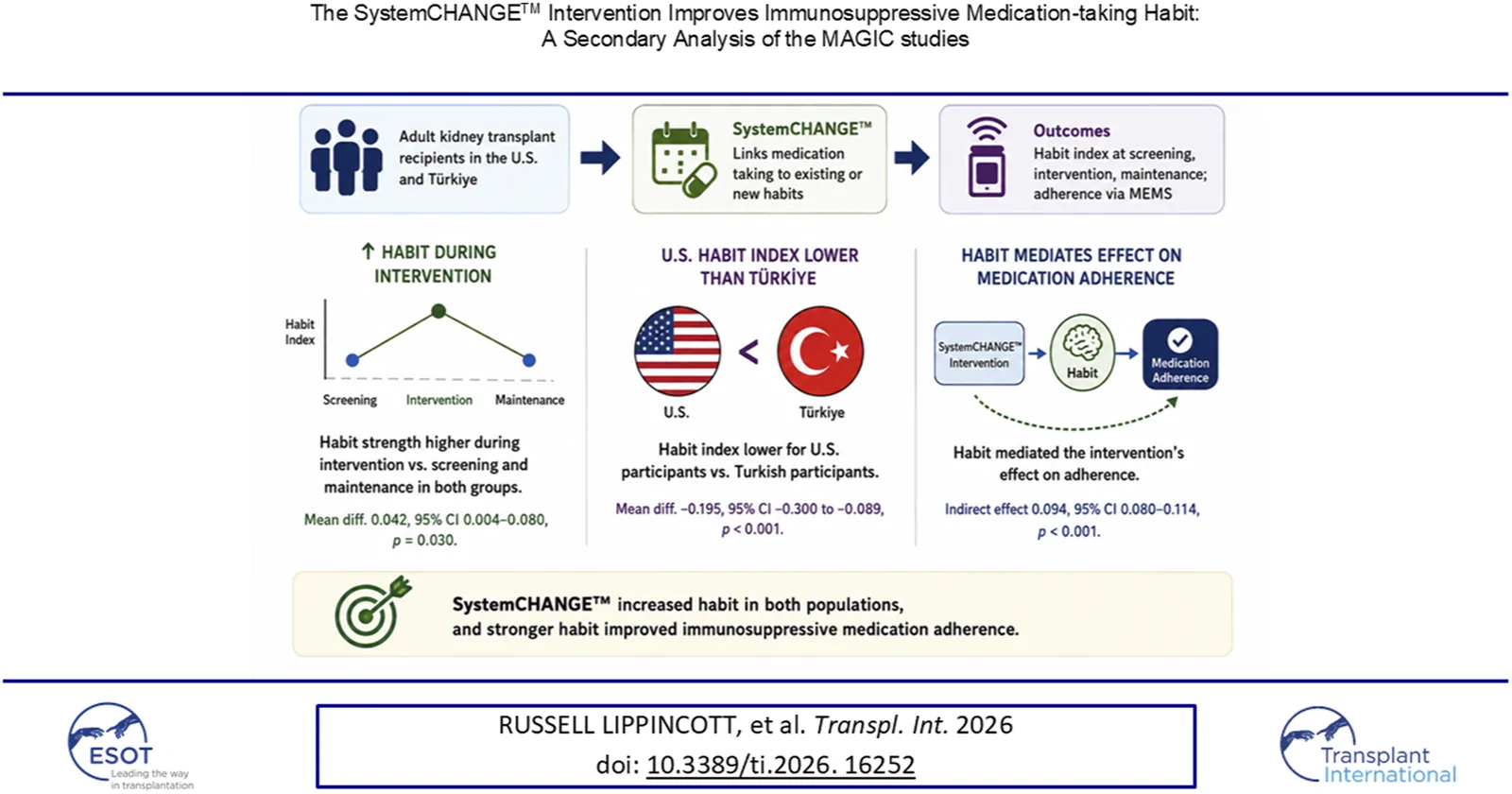

## Introduction

Health behavior change interventions have traditionally focused on motivation and intention with mixed results, particularly with difficult to change behaviors such as medication adherence. Such studies ignore routines and habits, which may account for their marginal effectiveness for individuals with acute and chronic illnesses [1–4] and equivocal findings for adult kidney transplant recipients [5].

Habit is critical in changing and maintaining healthy behaviors. Approximately 43% of daily behaviors are performed out of habit [6]. Habits are defined as automatic impulses to engage in a behavior in the presence of conditioned contextual cues. Habit formation is the process by which a behavior becomes automatic or habitual through regular repetition. Repetition of a behavior in a consistent context progressively leads to increased automaticity with which the behavior is performed when the situation is encountered [7, 8]. After some time, the automaticity of the behavior reaches a plateau and becomes a habit.

Duhigg theorized the “habit loop” to explain the process by which a habit is formed. The cue occurs first and “tells your brain to go into automatic mode and which habit to use”. [9], p.19 The routine happens next. It is the “physical, mental or emotional behavior that follows the cue”. [9], p.19 Finally, the reward occurs which “helps your brain figure out if this particular loop is worth remembering for the future”. [9], p.19 The loop becomes more automatic over time until a habit is created.

For a health behavior habit to form, it takes about 2 months, but the time is variable, with some habits taking nearly a year to form and become automatic [10]. Factors that positively influence habit formation include habits selected by individuals themselves, more frequent habit practice, morning habits, habits that are more enjoyable, stability of the context in which the habit takes place, and planning specifically for implementation of the habit [10].

The association between habit and medication adherence is clear. In a systematic review of 11 studies, using self-report of habit and behavior automaticity, habit strength was strongly associated with medication adherence in 91% of the studies [11]. Recently, habit strength has been measured using more rigorous objective measures instead of self-report. In a secondary analysis of 108 studies and 15,818 participants, most of whom were being treated for hypertension and osteoporosis, medication adherence was associated with lower daily or weekly medication timing variability, so stronger habit strength [12]. Additionally, habit strength accounted for over 30% of the variability in medication adherence. Likewise, in 79 patients with type 2 diabetes receiving oral medications, higher habit strength using an objective measure, was significantly associated with medication adherence [13].

The Change Habits by Applying New Goals and Experience (SystemCHANGE™) intervention, has propelled adherence interventions in a new direction [14, 15]. This is a secondary analysis of the US and Türkiye MAGIC study data, which sought to examine 1) differences in habit strength between the SystemCHANGE™ intervention group and the attention control group, as well as demographic predictors of this difference, 2) differences in habit strength between the two countries, and 3) habit as a mediator of the relationship between the SystemCHANGE™ intervention and immunosuppressive medication adherence, using a new objective measure of habit called the “habit index”, in adult kidney transplant recipients.

## Materials and methods

### Design

This is a secondary analysis of the MAGIC (ClinicalTrials.gov identifier NCT02416479) and MAGIC Türkiye (ClinicalTrials.gov identifier NCT06106854) studies’ data. The designs were single-blinded (participants), two‐arm randomized controlled trials using repeated measures [14, 15]. Immunosuppressive medication intakes during the three-month screening phase were recorded using electronic monitoring. Immunosuppressive medication adherence during screening was quantified by computing the agreement of recorded intakes with the prescribed number and timing of intakes. After the screening phase, participants with an immunosuppressive medication adherence score of ≥85% exited the studies and those with a score of less than 85% were given the opportunity to enter the intervention phase of the studies where they were randomized to either the SystemCHANGE™ intervention (treatment) or the attention control intervention (control). Electronic monitoring continued in both groups. The 85% cut point was established with adult kidney transplant recipients using a cluster analysis approach where data from those who were adherent with morning and evening immunosuppressive medication doses had an electronic medication monitoring adherence score ranging from 85% to 100% [16]. Additionally, immunosuppressive medication nonadherence below 85% has been shown to impact clinical outcomes for adult kidney transplant recipients [17]. All participants received standard care. The attention control intervention delivered over 6 months included monthly receipt of an educational brochure addressing healthy living with a kidney transplant. After the six-month intervention phase a six-month maintenance phase followed during which there was no intervention. Electronic monitoring of both groups continued. The objective of the maintenance phase was to observe if the effects of the intervention remained once the intervention stopped.

The SystemCHANGE™ intervention is based on socio-ecological theory [18] and process improvement methods [19]. In an iterative process using a plan-do-check-act improvement process, patients create a ‘personalized system solution’ by embedding their medication-taking in existing daily routines, evaluating the solution’s effect on consistent medication-taking guided by objective medication adherence data and an interventionist, then revising the solution as necessary and continuing the cycle.

### Setting and sample

For the MAGIC US study, participants were recruited from five transplant centers in the midwestern and southern US. For the MAGIC Türkiye study, participants were recruited from a transplant center in Izmir, Türkiye. The inclusion criteria were: individuals 18 years of age or older who had received a kidney‐only transplant; who self‐administered at least one prescribed immunosuppressive medication taken twice daily with a functioning kidney transplant; who were not hospitalized; had no diagnosis that would immediately shorten the lifespan; had access to a telephone; had the ability to speak, hear, and understand English or Turkish; were able to open an electronic monitoring cap on a medication bottle; had agreement from the transplant physician and nephrologist to participate; and were not cognitively impaired. In both studies, all participants who met the study inclusion criteria were invited to be in the study and screened for 3 months for adherence. For the US study, 281 individuals were excluded due to high adherence while 130 participants with medication non-adherence were invited to be in the intervention; 41 of these declined and 89 were randomized. Five participants dropped out from the study during the intervention period. For the Türkiye study, 16 were excluded due to high adherence while 60 participants with medication non-adherence were invited to be in the intervention; 18 of these declined/were unable to continue in the study and 42 were randomized with no attrition during the intervention phase.

### Instruments

The following variables were collected at baseline: gender, age, relationship status, education level (as a 3-level categorical variable: primary school, secondary school, and college or higher), employment status, etiology of kidney disease, and type of transplant (from living or deceased donor).

Immunosuppressive medication adherence was measured using the Medication Event Monitoring System ([MEMS®], AARDEX Group, Belgium) which includes a bottle cap with a microprocessor that records the occurrence and time of each bottle opening [20].

Habit index was computed from the objective medication adherence data using a recently developed habit index [13], quantifying week-by-week consistency in the pattern of medication intakes. This habit index was previously shown to correlate with a widespread, self-reported habit metric [13]. Computation of the objective habit index is located in the Supplementary Material. The habit index was computed for each participant during the entire 3-month screening, during each month of 6-month intervention and during each month of the 6-month maintenance period. The habit index ranges between 0 and 1, with 0 corresponding to taking medication at systematically different times (more than 2 h apart) from 1 week to the other, and 1 corresponding to a participant having a perfectly consistent medication intake pattern from week to week, e.g., taking the medication at 10:47 am every Monday, at 2:59 pm every Tuesday, not taking a dose on Wednesdays, etc.

### Procedure

For the MAGIC US study, the Institutional Review Board determined the present secondary analysis of the MAGIC US study data was exempt (Project number 2095804). For the MAGIC Türkiye study, the Institutional Review Board (Project number 2023/27-02) approved the study. Habit strength and immunosuppressive medication adherence data were extracted by a data analyst from the medication intake data.

### Data analysis

Demographic data were characterized using descriptive statistics. Chi-squared tests were used to compare count data between countries, and a Student’s unpaired t-test was used to compare age and baseline habit between the two populations.

A mixed linear model with random intercepts was used to model habit strength, measured at several timepoints, as a function of the following covariates: habit during screening, treatment group, phase in the study (intervention or maintenance), country, gender, age, and type of transplant (from living or deceased donor). Interaction terms between group, phase, and country were also modelled. These variables were selected *a priori* for inclusion in the model. Addition of more demographic variables in the model was investigated using a bidirectional forward stepwise approach and likelihood ratio tests, as detailed in the Supplementary Material. This procedure did not result in including additional demographic variables in the model.

A mediation analysis was performed to investigate whether habit was a mediator of the effect of the intervention on medication adherence. Medication adherence, more precisely its implementation component [21], was quantified as the proportion of days with exactly two immunosuppressive medication intakes. The mediation analysis used several regressions to estimate the direct effect of habit on medication adherence and its indirect effect through the intervention. The previously selected demographics were included in these regression models. Computations were performed using Python version 3.7.3 [22]. Further details on statistical methodology are provided as Supplementary Material.

## Results

Data from the MAGIC US study included 84 participants with mean age 51.6 years (standard deviation [SD] 10.6 years), with 58.3% (n = 49) male. The data from the MAGIC Türkiye study included 42 participants with mean age 46.1 years (SD 13.2 years), with 71.4% (n = 30) male. Table 1 delineates the overall and country-specific sample demographics. The two populations are different in several aspects: age, education, employment status and type of transplant.

**TABLE 1:** Participant demographics (n = 126).

Variable	Overall n, % or mean, ±SD	United States (n = 84)	Türkiye (n = 42)	p-value for equality
Gender Male Female	79 (62.7) 47 (37.3)	49 (58.3) 35 (41.7)	30 (71.4) 12 (28.6)	0.152
Age in years	49.7 ± 11.7	51.6 ± 10.6	46.1 ± 13.2	0.022
Relationship status Living with someone Single	75 (59.5) 51 (40.5)	48 (57.1) 36 (43.9)	27 (64.3) 15 (35.7)	0.441
Education Primary school Secondary school College or higher	23 (18.3) 59 (46.8) 44 (34.9)	5 (6.0) 54 (64.3) 25 (29.7)	18 (42.9) 5 (11.9) 19 (45.2)	<0.001
Employment status Retired Unemployed/Housewife Disabled/self employed/other Working full or part-time	21 (16.7) 15 (11.9) 31 (24.6) 59 (46.8)	9 (10.7) 7 (8.3) 31 (36.9) 37 (44.0)	12 (28.6) 8 (19.0) 0 (0.0) 22 (52.4)	<0.001
Etiology of kidney disease Hypertension Polycystic kidney disease Diabetes mellitus CKD/Other/Unknown/ Missing	45 (35.7) 14 (11.1) 23 (18.3) 4 (34.9)	30 (35.7) 10 (11.9) 11 (13.0) 31 (39.4)	15 (35.7) 4 (9.5) 12 (28.6) 11 (26.2)	0.193
Type of transplant Cadaveric/deceased donor Living donor	79 (62.7) 47 (37.3)	62 (73.8) 22 (26.2)	17 (40.5) 25 (59.5)	<0.001
Baseline habit	0.517 ± 0.232	0.400 ± 0.146	0.764 ± 0.180	<0.001

**FIGURE 1:**
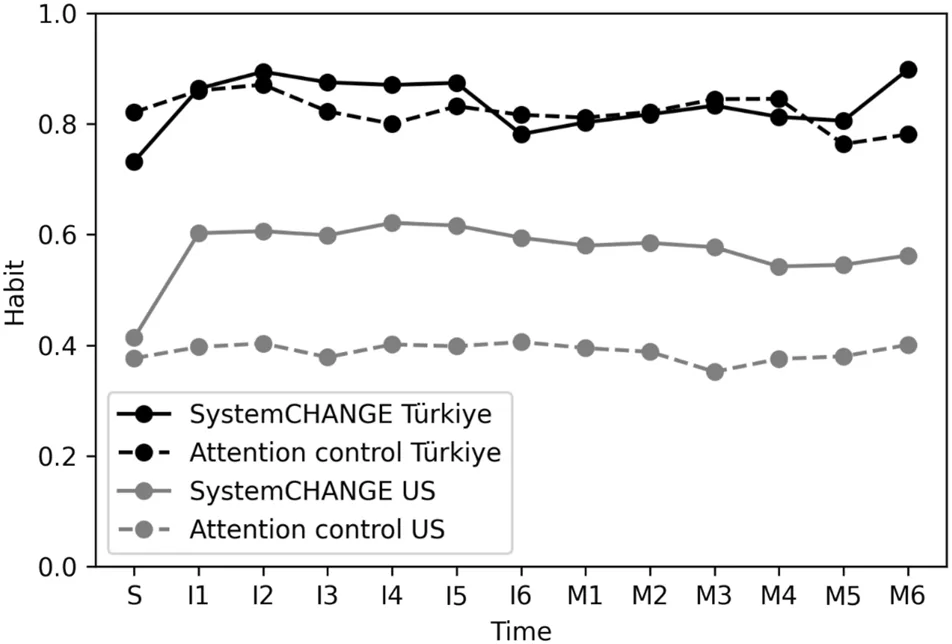
Mean habit strength by month for SystemCHANGE and Attention control for both US and Turkiye. Note: S, Screening; I=Intervention; M, Maintenance.

Figure 1 shows the evolution of mean habit strength among the two groups (intervention versus attention control) among the two studies (US and Türkiye). The underlying data are provided in a Supplementary Table. For participants in the intervention groups for both countries, habit strength was higher during the SystemCHANGE™ intervention than during screening and maintenance (regression coefficient for interaction between group and study phase = 0.042, 95% CI: 0.004 to 0.080, p = 0.030, with reference group being control and reference phase being maintenance). Participants from the US had a lower habit index overall when compared to Türkiye (regression coefficient for country being US = −0.195, 95% CI: −0.300 to −0.089, p < 0.001). Habit in US participants seems to remain higher even during the maintenance phase (regression coefficient for interaction between country and group = 0.111, 95% CI: 0.002 to 0.221, p = 0.047 with reference group being control and reference country being US). Finally, older participants had a slightly higher habit index (regression coefficient = 0.002, 95% CI: 0:000 to 0.005, p = 0.038). No other demographic variable had a statistically significant impact.

The mediation analysis demonstrated that habit mediated the effect of intervention on medication adherence. The SystemCHANGE™ intervention had a significant effect on adherence (total effect = 0.215, 95% CI: 0.192 to 0.238, p < 0.001). The direct effect of the intervention on adherence was 0.121 (95% CI: 0.102 to 0.140, p < 0.001). The indirect effect of the intervention on adherence (through improving habit) was 0.094 (95% CI: 0.080 to 0.114, p < 0.001), meaning that approximately 43% of the effect of the intervention is mediated by habit.

## Discussion

This is the first study to show that habit strength, measured using a new objective habit metric, was statistically significantly higher when participants were receiving the SystemCHANGE™ intervention, compared to those receiving an attention-control intervention. The SystemCHANGE™ intervention’s impact on habit was immediately effective because the participants implemented the SystemCHANGE™ at the beginning of the intervention when the intervenor and the participant collaborated to explore habits around desired medication taking time. This included identifying important people for the medication taking process, examining the participant’s life routines, habits and life cycles, and identifying and initiating a SystemCHANGE™ personalized system solution. For example, these solutions included placing medication next to regularly occurring habits such as near the coffee pot when making coffee, near the toothbrush when brushing teeth, near the television remote control for watching favorite shows, or near pet medications when pets received their medications. Because these solutions embedded medications in existing habits and were implemented at the beginning of the intervention, habit strength and consequently, medication adherence, immediately improved and were maintained [14, 23, 24]. As such, this provides evidence for future testing of reduced ‘doses’ of the SystemCHANGE™ intervention or perhaps providing periodic boosters.

These findings are consistent with a recent systematic review of 11 studies including people taking medications for asthma, diabetes, hypertension, cystic fibrosis, psoriasis and contraception that found habit strength was strongly correlated with medication adherence, and stronger habit was associated with higher medication adherence rates [11]. These findings are also congruent with the recent study by Phillips et al. where the same objective habit metric was used to demonstrate an association between habit strength and medication adherence in adults with type 2 diabetes during a 1 month period [13].

This study is also the first to explore habit strength across two different countries, and therefore, cultural groups finding country-level differences in medication-taking habit during screening, during the SystemCHANGE™ intervention and the maintenance period between the US and Türkiye. These differences may originate from the different populations included in the two studies. The finding that habit strength was higher in the younger Turkish sample compared to the older US participants contrasts with the findings that older aged individuals, even with some cognitive decline, had higher medication taking automatized routines compared to healthy adults [25]. The routines and habits established in older individuals have been viewed as protective for medication adherence.

Other, more important differences than age between the two populations could explain the differences. The influence of the Turkish sample having significantly more living donor kidney transplants than the US sample on habit strength and thus immunosuppressive medication adherence could be explained by the findings that Turkiye has the highest rate of living-donor transplantation worldwide [26]. Moreover, nearly 80% of living-donor transplantations are performed using organs donated by first- or second-degree relatives. This unique characteristic may influence post-transplant outcomes, as family-member donors are often more actively involved in the recipient’s care process. Living donors may monitor the recipients of their kidneys in matters such as taking medications [27]. This may have contributed to the high level of medication adherence among recipients. Such involvement may enhance the recipient’s awareness of the donation process and foster a greater sense of responsibility toward protecting the transplanted organ, thereby promoting better adherence to medical recommendations and long-term graft care.

These results confirm the hypothesized effect of the SystemCHANGE™ intervention. Indeed, this intervention is designed to link medication-taking to a patient’s established routines, to support improving medication-taking habit. The results of the present work demonstrated that habit was a mediator of the positive effect of the intervention on medication adherence.

### Strengths and limitations

This study’s follow up of 6 months for the intervention period and 6 months for the maintenance period are much longer than previous habit strength studies [13]. The SystemCHANGE™ intervention taps into reliable personal systems for linkage of medication taking routines to habit so that medication taking happens at the same time every day without relying on intention or memory. Consequently, habit strength and thus medication adherence is sustained over long periods of time [14].

The original MAGIC studies aimed at testing whether the SystemCHANGE™ intervention improved medication adherence. Studies on behavioral interventions, by design, suffer a self-selection bias, as participants who may benefit the most from an intervention are also more likely to decline participation in the study [28].

The size of the US sample was determined through a power analysis aimed at detecting an effect of the SystemCHANGE™ intervention on medication adherence. This analysis is reported in the original article [14]. On the other hand, the Turkish sample was small, which might have prevented detecting an effect of the intervention during the maintenance period. This could limit the generalizability of the findings to the population. Additionally, important structural differences between the populations such as age, education level, employment status, and type of transplant donor may introduce confounding and limit the interpretation of country-level differences. The finding that the Turkish sample was younger, less educated, retired/less disabled, and received more living donor kidney transplants could have influenced the results and therefore, country-level differences should be interpreted with caution.

This analysis did not examine other variables that may influence immunosuppressive medication taking habit strength such as time of day during which the habit occurred and stability of the context in which the habit took place [10]. Additionally, depression and anxiety, two factors that may influence habit were also not studied [29].

The reference value of the habit index was computed over the entire 3-month screening period, to replicate the methodology that was used for the computation of the medication adherence score in the original MAGIC studies. However, habit (and medication adherence) may have changed during the 3-month screening period, because of the introduction of electronic monitoring of adherence causing a Hawthorne effect. The effect of the introduction of electronic monitoring on adherence is unclear [16, 30, 31] and, to the best of the authors’ knowledge, the effect of the introduction of electronic monitoring on habit has not been studied.

Finally, because this work is a secondary analysis that was not prespecified in the design of the original MAGIC studies, it carries the associated limitations. Deciding upon this analysis *a posteriori*, once the trial results were already available, introduces a potential risk of analytical bias, meaning these findings should be considered exploratory.

### Clinical implications

These findings have important clinical implications. Briefly assessing habits of kidney transplant recipients around the prescribed times of immunosuppressive medication taking could provide actionable strategies for linking medication taking to existing routines therefore establishing good medication taking habits. Additionally, conversations between the provider and the patient should happen early in the post-transplant period so positive medication taking habits can be established as soon as possible after transplant.

### Future research

Further trials are planned to examine whether these results are replicable in those with other chronic conditions such as chronic kidney disease and breast cancer using the SystemCHANGE™ intervention, the habit strength index, and objectively measured medication adherence using the MEMS®. Additionally, multi-level influences on habit strength should be explored, including the link between demographic variables and habit strength. Finally, the 6-month SystemCHANGE intervention involved monthly “checks” with participants collaboratively reviewing their medication adherence data and assessing whether the SystemCHANGE solution was continuing to be effective. The intervention was immediately effective within the first month with a slight drop in habit strength between months 5 and 6. Consequently, testing a 3 and 6 months SystemCHANGE “booster” seems like the next logical step in this line of research.

## Conclusion

This study’s results support that objectively measured habit strength is higher at the completion of a six-month SystemCHANGE™ intervention when compared to a six-month attention-control intervention in two culturally diverse samples of adult kidney transplant recipients. This positive impact on habit strength is linked to a positive impact on medication adherence.

## Data Availability

The data analyzed in this study is subject to the following licenses/restrictions: The dataset is available upon contacting the corresponding author and with IRB approval. Requests to access these datasets should be directed to Cynthia Russell Lippincott russellc@umkc.edu.
