# Supplementary material for: The SystemCHANGE™ intervention improves immunosuppressive medication-taking habit: a secondary analysis of the MAGIC studies
Source: Transpl Int. 2026 Sep 1;39:16252. doi: 10.3389/ti.2026.16252 (PMC13574724; doi:10.3389/ti.2026.16252)
Supplement: Supplementary file 1 [file Supplementaryfile1.docx]

# Computation of the objective habit index

To compute the objective habit index used in this study, medication intakes are represented in a two-dimensional array with 24 rows and one column for each day of follow-up. This array, denoted A, is initially filled with zeros and is iteratively populated as follows: for each medication intake

1. Let d denote the index of the day on which this medication intake occurred
2. Let h and m denote the hour and minutes on which this medication intake occurred
3. Let A[h, d] = A[h, d] + 1 – m/60
4. If h < 23, let A[h + 1, d] = A[h + 1, d] + m/60
5. If h = 23, let A[0, d + 1] = A[0, d + 1] + m/60

When all intakes are processed, the resulting array can be represented in two dimensions as a greyscale picture on which darker areas correspond to times of medication intakes. Two examples are provided in the figure below.

The habit index quantifies the week-to-week consistency of medication intakes. To do so, each element A[i, j] of the array is multiplied with its counterpart one week ahead, A[i, j + 7]. If intakes occurred around the same time on days j and j + 7, the product A[i, j] A[i, j + 7] will be close to 1. Otherwise, the product will be close to zero.

All these individual products are then summed all together for all days and hours, and the result is divided by the norm of A, so that the habit index ranges between 0 and 1:

Habit index = $\frac{\sum_{i,j} A\left[ i,j \right]A\left[ i, j+7 \right]}{\sum_{i,j} A\left[ i,j \right]^{2}}$

If the habit index equals 0, this means that no intake occurred at the same time (in a 2-hour window) from one week to the other. If the habit index equals 1, it means that the intake pattern repeats itself perfectly from week to week. As an example, the figure below provides a visualization of A for two different participants. The participant on the left has a habit index equal to 0.13 and the participant on the right, 0.76.
