# Supplementary material for: The SystemCHANGE™ intervention improves immunosuppressive medication-taking habit: a secondary analysis of the MAGIC studies
Source: Transpl Int. 2026 Sep 1;39:16252. doi: 10.3389/ti.2026.16252 (PMC13574724; doi:10.3389/ti.2026.16252)
Supplement: Supplementary file 2 [file Supplementaryfile2.docx]

# Appendix: statistical methodology

## Missing data handling

Missing data for employment category and etiology of kidney disease were coded as a separate category. There was no missing data for other baseline characteristics. Missing data for baseline habit (5 participants) and age (1 participant) were imputed with multiple imputation by chained equations (MICE) using the Python package miceforest. Using this approach relies on a missing-at-random hypothesis, where missing data can be accounted for by variables with no missing data. We believe this hypothesis is reasonable given, for instance, the link between country and age and between country and baseline habit.

Two Turkish participants had no MEMS data and were removed from the analysis.

Missing data for habit after baseline were dropped. This resulted in removing another Turkish participant from the analysis, because they had no habit data after baseline.

The final dataset comprised 123 participants, with 1,355 monthly measures of habit.

## Variable selection procedure

The following variables were available in the dataset:

- Baseline habit,
- Country,
- Gender,
- Age,
- Education level,
- Etiology of kidney disease,
- Employment category,
- Relationship status,
- Type of transplant (from deceased or living donor),
- Treatment group (intervention or control),
- Phase in the study (during intervention or maintenance).

Domain experts were consulted to determine which demographic variables should be included a priori in the model. They selected the following demographic variables as relevant: country, gender, age, and type of transplant.

In the present study, the US sample is twice the size of the Turkish sample. To avoid finding treatment group and/or study phase effects which are mostly driven by US participants, we also included a priori the following predictors in the model:

- Treatment group,
- Interaction between country and treatment group,
- Interaction between phase in the study and treatment group,
- Interaction between country, phase in the study, and treatment group.

This allowed us to separately estimate the effect of the country and the effect of treatment group and/or study phase.

As a second step, the four remaining demographic variables (education level, etiology of kidney disease, employment category and relationship status) were tested for inclusion using a forward bidirectional stepwise procedure. Each variable was tested individually: a likelihood ratio test was performed to compare the model with the additional variable to the initial model. The variable corresponding to the test with the lowest significant p-value was included. Then, each variable included up to now was individually tested for removal by testing models with and without this variable. The process was repeated until the no new included or removed variable yielded a significant p-value for the likelihood ratio test.

This method was chosen to minimize the number of additional variables included in the model. After applying it on the dataset, no additional demographic variable ended up being included in the model.

## Variance inflation factor

Correlation between explanatory variables included in the final model was investigated using variance inflation factor analysis. The variance inflation factor for each variable is:

- Baseline habit: 2.52
- Country: 2.60
- Gender: 1.05
- Age: 1.16
- Type of transplant: 1.18
- Treatment group (control or intervention): 1.04
- Phase in the study (intervention or maintenance): 1.00

The values of the variance inflation factors for all variables were low, well below the conservative threshold of 5. As a consequence, this analysis did not highlight any important correlation between model variables.

## Final model

The following table presents the results of the mixed model with random intercepts fitted to the data. Explanatory variables with a p-value < 0.05 are highlighted in bold.

|  | Coefficient | Lower CI | Upper CI | p-value |
| --- | --- | --- | --- | --- |
| **Intercept** | **0.265** | **0.073** | **0.456** | **0.007** |
| **Baseline habit** | **0.539** | **0.379** | **0.698** | **< 0.001** |
| During intervention phase | 0.012 | -0.005 | 0.030 | 0.165 |
| Group is intervention | 0.041 | -0.051 | 0.132 | 0.381 |
| **Country is US** | **-0.195** | **-0.300** | **-0.089** | **< 0.001** |
| **During intervention phase and group is intervention** | **0.042** | **0.004** | **0.080** | **0.030** |
| **Group is intervention and country is US** | **0.111** | **0.002** | **0.221** | **0.047** |
| During intervention phase and group is intervention and country is US | -0.011 | -0.050 | 0.029 | 0.602 |
| Gender is female | -0.015 | -0.066 | 0.036 | 0.562 |
| **Age (years)** | **0.002** | **0.000** | **0.005** | **0.038** |
| Transplant is from a living donor | 0.001 | -0.054 | 0.055 | 0.981 |

### Model checks

Residuals of the fitted model were analyzed using a histogram, a Q-Q plot, and a plot of residuals versus fitted values. These plots highlighted no skew in the residuals.

## Mediation analysis

A mediation analysis was performed to assess whether habit acts as a mediator between the SystemCHANGE^TM^ intervention and adherence. Previously selected demographic variables were included in the mediation analysis. The results are presented in the table below.

| **Path** | **Coefficient** | **p-value** | **Lower CI** | **Upper CI** |
| --- | --- | --- | --- | --- |
| Habit ~ intervention | 0.141 | < 0.001 | 0.120 | 0.163 |
| Adherence ~ habit | 0.761 | < 0.001 | 0.717 | 0.805 |
| Total effect | 0.215 | < 0.001 | 0.192 | 0.238 |
| Direct effect | 0.121 | < 0.001 | 0.102 | 0.140 |
| Indirect effect | 0.094 | < 0.001 | 0.080 | 0.114 |

The results demonstrate a statistically significant indirect effect of the intervention on adherence through habit strength (coefficient is 0.094). These results confirm that habit acts as a mediator between the SystemCHANGE^TM^ intervention and adherence, accounting for approximately 43% of the total effect.

## Sensitivity analyses

To confirm the validity of the results, two sensitivity analyses were performed. First, a random effects model with random slopes and random intercepts was tested. Second, the original analysis was repeated on a balanced sample between US and Türkiye.

### Model with random slopes and random intercepts

The following table presents the results of the model fitted with random slopes and random intercepts. Importantly, the variance between participants for the random slope was 0.000, suggesting that including random slopes in the final model was not necessary.

|  | Coefficient | Lower CI | Upper CI | p-value |
| --- | --- | --- | --- | --- |
| **Intercept** | **0.310** | **0.107** | **0.512** | **0.003** |
| **Baseline habit** | **0.531** | **0.369** | **0.692** | **< 0.001** |
| During intervention phase | 0.008 | -0.016 | 0.032 | 0.528 |
| Group is intervention | 0.038 | -0.061 | 0.137 | 0.453 |
| **Country is US** | **-0.194** | **-0.301** | **-0.088** | **< 0.001** |
| **During intervention phase and group is intervention** | **0.054** | **0.002** | **0.106** | **0.044** |
| Group is intervention and country is US | 0.111 | -0.005 | 0.228 | 0.061 |
| During intervention phase and group is intervention and country is US | -0.024 | -0.078 | 0.031 | 0.395 |
| Gender is female | -0.023 | -0.075 | 0.028 | 0.376 |
| Age (years) | 0.002 | -0.000 | 0.004 | 0.118 |
| Transplant is from a living donor | -0.009 | -0.065 | 0.048 | 0.766 |

### Under-sampling the US population

To assess the impact of the sample imbalance between US and Türkiye, we performed a sensitivity analysis in which we randomly subsampled the US population (84 participants) to match the size of the Turkish population (39 participants). A random effects model with random intercepts was then fit to this balanced population. This analysis was repeated 100 times, and the resulting parameter values were collected. The summary statistics of the parameter values are presented in the following table. Variables displayed in bold exert the same effect across the 100 repetitions.

|  | Median | Minimum | Maximum |
| --- | --- | --- | --- |
| **Intercept** | **0.235** | **0.080** | **0.415** |
| **Baseline habit** | **0.571** | **0.432** | **0.692** |
| During intervention phase | 0.015 | -0.006 | 0.048 |
| **Group is intervention** | **0.049** | **0.035** | **0.068** |
| **Country is US** | **-0.171** | **-0.241** | **-0.076** |
| **During intervention phase and group is intervention** | **0.050** | **0.018** | **0.071** |
| Group is intervention and country is US | 0.099 | -0.002 | 0.187 |
| During intervention phase and group is intervention and country is US | -0.023 | -0.084 | 0.029 |
| Gender is female | -0.021 | -0.072 | 0.017 |
| **Age (years)** | **0.002** | **0.001** | **0.004** |
| Transplant is from a living donor | -0.008 | -0.066 | 0.068 |
